# Sir Benjamin William Rycroft OBE (1902–1967): British ophthalmologist and pioneer in corneal surgery

**DOI:** 10.1177/09677720231186416

**Published:** 2023-08-23

**Authors:** Josh Wilcox, Maxwell Cooper

**Affiliations:** 12190 Department of Primary Care and Public Health, Brighton and Sussex Medical School, Brighton, UK

**Keywords:** Rycroft, ophthalmology, Second World War, Sussex, keratoplasty, cornea

## Abstract

An unsung hero of British ophthalmology is the largely forgotten Sir Benjamin Rycroft (1902–1967). This paper will discuss and analyse the undervalued career of this great man. Upon graduating from medical school, Rycroft became a General Practitioner. Rycroft then decided to train to become an ophthalmologist. Rycroft began his ophthalmology career in 1930s London focusing on the new ground-breaking surgery of keratoplasty (corneal grafting) before serving with distinction in the medical corps during the Second World War. He is chiefly remembered for his work after the war at the Queen Victoria Hospital in East Grinstead, Sussex, where he worked with renowned plastics surgeon Archibald McIndoe. During his time, there Rycroft became globally recognised for his skill in keratoplasty and started a campaign which radically changed the legal framework behind organ donation in the UK. Despite few knowing of him today, Rycroft is undoubtedly one of the most influential British ophthalmologists of the past century. He was for decades seen as one of the world's leading practitioners of keratoplasty and established a unit which restored sight to wounded veterans. His greatest achievement lies in his organ donation reform, which started the process of allowing organ donation to be carried out on a nationwide scale for the first time.

## Introduction

**Figure 1.**
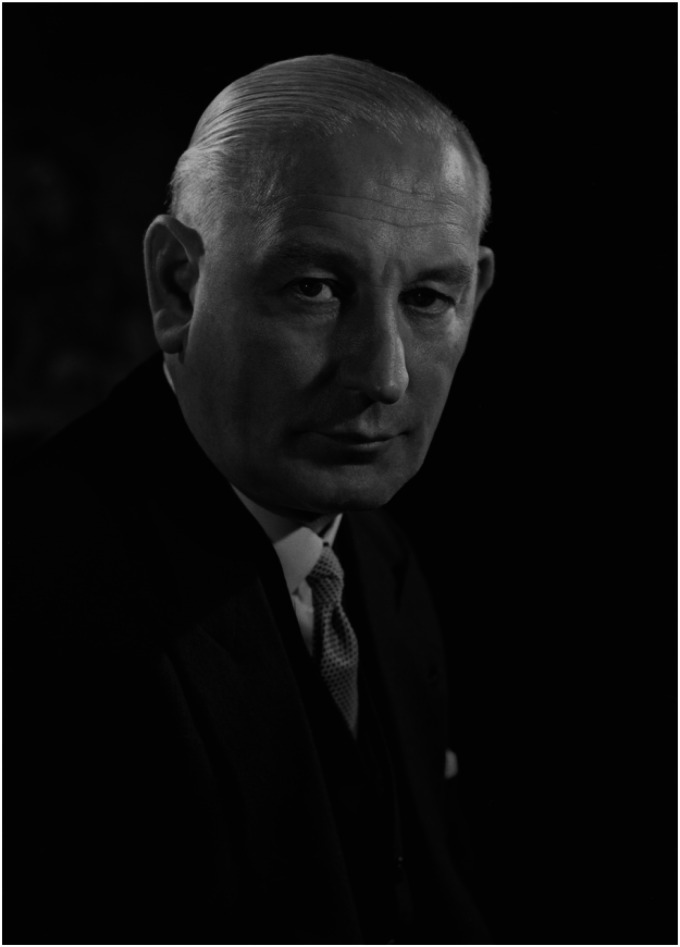
Sir Benjamin William Rycroft by Walter Bird, July 1960, courtesy and copyright of the National Portrait Gallery, London.

The UK has a rich history in ophthalmology from the founding of the world's first eye hospital (Moorfields) to the invention of the intraocular lens by Sir Harold Ridley (1906–2001) which revolutionised cataract surgery.^1^ A high point of British ophthalmology came in the mid-twentieth century with the emergence of pioneering ophthalmologists shaped by the world wars and their aftermaths. One of these was Sir Benjamin Rycroft (1902–1967) who spent his ophthalmological career advancing the recent surgical innovation of corneal grafting (keratoplasty). Keratoplasty is an operation to remove all or part of a patient's damaged cornea and replace it with a new healthy cornea from a donor.^2^ Rycroft's legacy, however, lies not only in keratoplasty as he (unwittingly) set in motion the wider process of organ donation reform which now means thousands of patients can receive transplant surgeries each year in the UK (Figure 1).

## Early life (1902–1919)

Benjamin William Rycroft was born on the 16^th^ of August 1902 in the Yorkshire village of Armley.^3^ His father was John Thomas Rycroft (1869–1955) and his mother was Annie Elizabeth Hudson (1876–1960).^4,5^ Benjamin was an only child and named after his paternal grandfather Benjamin Rycroft (1813–1878) who passed away when John was only nine.^6^ John and Annie had married in their hometown of Bradford in 1898 before moving to the nearby parish of Armley in Leeds where Benjamin was born and spent his early childhood.^5,7^

Today, Armley is an urban district in the west of Leeds situated just under a mile from the Leeds city centre.^8^ Like much of Leeds, Armley was right at the heart of the Industrial Revolution and for most of its history has been and remains a working-class area. In 1788, the mills in Armley were bought and expanded to become the then-largest woollen mill in the world with most employees living nearby in the terraced houses of Armley.^9^ These mills were still in operation by the time that Benjamin lived in Armley as they only shut down in 1971.^10^ This closure formed part of a major redevelopment programme that created much of modern Leeds.^11^ Benjamin's formative years would have been in a grey, smoky and harsh industrial neighbourhood.

A way that Benjamin sought shelter from this bleak environment was through the local parish church of St Bartholomew's.^12^ St Bartholomew's is one of two churches in Armley and best known for its large and impressive organ built by famed German organ builder Edmund Schulze (1824–1878) and installed in the church in 1879.^13^ Benjamin fell in love with the organ and was able to play well enough to do so in many of the churches that he would attend throughout his life.^12^ The manual dexterity to play such a complex instrument suggests Benjamin's musical skill may have proved helpful later in life as a surgeon. His passion for the church led him to give serious consideration to joining the priesthood.^12^

By 1911, Benjamin's father John had decided to return to Bradford where they moved to the suburban village of Great Horton.^4^ It is unclear whether Annie joined them in this move which could suggest that John and Annie potentially separated. The 1911 census lists John's profession simply as ‘cashier’ which reveals a likely banking or retail setting.^4^ However, John donated money to the local primary school in Lidget Green to pay for 20% of students’ fees which suggest a moderately sized income and a philanthropic heart.^13^ Great Horton was likewise another industrial mill town situated on the outskirts of Bradford on the road to Halifax.^14^ When the First World War broke out the twelve-year-old Benjamin was too young to volunteer yet old enough to appreciate its devastation. One account of someone who knew the young and confident Benjamin described him as being ‘a man of foresight, energy and ambition’.^15^

## Early adulthood 1919–1931

Benjamin was certainly ambitious and after the First World War ended Benjamin matriculated at the University of St Andrews in Scotland to study medicine in the autumn of 1919.^16^ Benjamin spent five years in the beautiful but windswept coastal Fife town before graduating in 1924 when he received his MBChB.^17^ As a medical student, he was duly honoured with winning the university medal in operative surgery which was a good harbinger of his future specialty. In 1924, he also received his Conjoint diploma.^18^ The conjoint diploma was a common ‘back-up’ alternative to university final examinations.^19^

Upon graduation, Benjamin decided to return to Bradford where he started his clinical career as a General Practitioner (GP).^18^ This would have given Benjamin a robust grounding in the medical profession and for the remainder of his life he always spoke fondly of General Practice and proud of all he learned in the role. He became widely known and well respected in Bradford as a GP.^20^ His surgery during this period was situated at 22 Bingley Road in the Bradford ward of Heaton.^21^ He spent most of the 1920s content in Bradford and it was during these years that he married Mary Elizabeth Rhodes.^22^

Mary was three years older than Benjamin as she was born on the 30^th^ of April in 1899 in the leafy village of Allerton, just outside of Bradford.^23^ Unlike Benjamin, she came from a Nonconformist background in contrast to his Anglican one and was also from a larger family with many siblings.^23^ Her father Fred Rhodes (1873–1959) was a prominent wool merchant and had premises in Armley where he would trade with the local mills. This suggests Benjamin and Mary had likely known each other since early childhood due to their Armley connection.^24^ In records from the 1920s, Benjamin's father John also starts to be listed as a merchant so he probably knew and traded with Fred Rhodes. This may offer further insight into John's mysterious income and how he could afford to send Benjamin to such a prestigious university.^22^

Benjamin and Mary wed on the 30th of November 1924 at St Barnabas Church in Heaton, close to where Benjamin practised.^22^ Benjamin had been living in Heaton before the marriage and Mary joined him there after the ceremony. Heaton was famously the childhood home of the esteemed playwright JB Priestley (1894–1984) and was considered by Priestley and many others to be one of the most respectable neighbourhoods in the city during the early half of the 20th century.^25^ Sadly, since the Rycrofts left Heaton, the area is today remembered due to its connection to the Yorkshire Ripper.^25^

The fact that the young couple was able to live in such an affluent neighbourhood reveals they were already financially secure. The couple were always close and throughout their marriage whenever Benjamin travelled abroad Mary made sure to always come as his companion. They would remain happily married until Benjamin's death and together had two loving sons. The eldest was Peter, born in 1928 in Bradford, and the youngest was Rodney who was born in 1935 once they had moved south.^26^ One constant throughout Benjamin's career was that no matter how hard his day had been at the hospital he always had a calm and happy home to return to.

During the latter half of the 1920s, Benjamin's focus switched from General Practice as he began the long and arduous journey to become a surgeon. He received his M.D. in 1928 and his Diploma in Ophthalmic Medicine and Surgery (D.O.M.S.) in 1929.^18^ His single-minded drive for success meant that during this period he would routinely travel for hours to London for studying before returning to Bradford to work in General Practice at the weekends.^18^ He often attended his teaching at St Bartholomew's Hospital at all sorts of unusual and unsociable hours to maximise learning opportunities yet allowing time to return to Bradford to see his family.^15^ It is serendipitous that Benjamin trained to be a surgeon in the hospital with the same name as that of the church where he learned the organ. The London medical elite may have been surprised by a Yorkshire GP deciding to train for surgery, but Benjamin definitely relished the attention. Some suggested Benjamin owed his ferocious appetite and thirst for learning to his tough industrial roots as well as his religious fervour. In 1931, Benjamin was finally awarded his Fellowship of the Royal College of Surgeons (F.R.C.S) in what was the culmination of over a decade's worth of intense studying.^18^

## Moving south and start of ophthalmological career 1931–1939

It was in 1931 that Benjamin took the momentous decision to move the family south to live in the Buckinghamshire village of Taplow on the edge of Maidenhead. A few years later, they moved again to live at the quaint and picturesque farm Bishop's Lodge in the Windsor suburb of Oakley Green.^3^ They did this so Benjamin could try and break through into the highly prestigious world of London ophthalmological hospitals. Leaving behind the familiarity of Bradford and their extended families must have been an upheaval for Mary and Peter too.^27^

Benjamin took his first job in London at St George's Hospital.^27^ Here, he was clinical assistant to Sir Stewart Duke-Elder (1898–1978) who was knighted not long after Benjamin arrived in 1933.^20^ Duke-Elder was a fellow St Andrew's alumnus so probably knew Benjamin already and likely had the greatest impact of anyone on Benjamin's career as an advisor, mentor and confidante. If Benjamin were to choose a mentor, then he could not have chosen any better than Duke-Elder. Duke-Elder was arguably one of the most distinguished ophthalmologists of the era and would treat the then-prime minister Ramsay MacDonald for glaucoma as well as being a Surgeon-Oculist to three monarchs: Edward VIII, George VI and Elizabeth II.^28^ Duke-Elder's greatest legacy is in the area of ophthalmological literature as the author of the classic seven-volume *Textbook of Ophthalmology* which he wrote over a span of 20 years. He would for many years be the leading editor of the *British Journal of Ophthalmology* and *Ophthalmology Literature*.^28^

He eventually progressed to working at Moorfields Hospital which was founded in 1805. It is unknown when Benjamin decided to pursue a career operating on the eye but his interest may have been cultivated at university. It was at Moorfields that Benjamin became fascinated with the problem of corneal transplantation.^12^ The first human-to-human corneal transplant had been performed in 1905 by the Austrian ophthalmologist Eduard Zirm (1863–1944).^29^ Zirm replaced a cornea of a local farmer who had suffered corneal damage from alkaline poisoning with one of a young child who had irreversible trauma to his eyes. The first successful British corneal transplant was carried out in 1930 by esteemed ophthalmologist Tudor Thomas (1893–1976).^30^ Thomas had initially demonstrated the procedure on a rabbit before attempting the human operation and only reported the case in 1933.^30^ This clinical problem would be one to which Rycroft would go on to devote most of his professional life.^12^ Benjamin fell in love with keratoplasty whilst it was still in its infancy but saw huge potential in the operation. The procedure has over time evolved from replacing the entire cornea to one which focuses on removing only the diseased corneal layers. Nowadays keratoplasty is the most successful transplantation carried out on the human body as the cornea lacks vasculature, so graft rejection is extremely rare.^2^

Research was a field that interested Benjamin greatly and in 1935, the same year that his son Rodney was born, he had his first paper published on corneal grafting.^12^ Benjamin described in a BMJ article an operation for corneal grafting in intricate and precise detail using a thorough case study.^31^ Another one of Benjamin's clinical interests was the ocular management of the autoimmune condition pemphigus. Pemphigus is a rare condition where the immune system attacks a patient's epidermis causing painful blisters all over the body.^32^ Benjamin wrote various journal papers over his career on the management of ocular blisters where he analytically discussed the different treatment options. His landmark 1934 paper on pemphigus helped establish his reputation as he was the first person to divide conjunctival pemphigus into two forms [acute and chronic]. Rycroft went on to explain how the treatment would then vary depending on the form.^32^

Despite only recently entering the world of ophthalmology, it did not take long for the industrious Benjamin to start making a name for himself. His devotion to the specialty along with his honesty, efficiency and kindness made him popular and well-known amongst his colleagues.^12^ His fervour for ophthalmological surgery around this time bore fruit in the form of prestigious awards: a Hunterian professorship and Leverhulme scholarship at the Royal College of Surgeons, a Lang Research Scholarship at Moorfields and the Middlemore Prize of the British Medical Association.^27^

He also began to be associated with more hospitals including the King's College Hospital Group, the Royal Northern Hospital near King's Cross, the King George Hospital in Ilford, the East Ham Memorial Hospital, the Royal Eye Hospital and the Rycroft's local Maidenhead Hospital.^27^ He also became a civilian consultant in ophthalmology to the Ministry of Aviation and an honorary consultant in ophthalmology to the Marylebone Cricket Club and the Zoological Society of London.^18^ His role at the Zoological Society gave Benjamin a unique opportunity to operate on tigers and horses among many other exotic animals. As a university examiner, he would routinely ask questions of a veterinary nature. An example question is what sized tracheal tube does an average adult female tiger require? The answer obviously is a number 15 Magill-type tracheal tube.^20^

Alongside clinical practice and academic studies, he also started to amass a large following of private patients who all became ardent admirers.^12^ Throughout his career; his private patients would remain notoriously loyal. He had premises on Harley Street and by the time of his death, he had over 15,000 individuals on his register.^12^ Benjamin's work attracted many of the British elite and his patients included the Queen Mother and Winston Churchill's wife Clementine who Benjamin treated in 1959.^12^

## The Second World War and its Aftermath

His ophthalmological career in London was interrupted when war broke out again in September 1939, but this did not hold Benjamin back as he was quick to sign up for the Royal Army Medical Corps.^27^ He was initially based at the 31^st^ General Hospital at Musgrave Park in Belfast, Northern Ireland, before serving with distinction in North Africa and finally in Italy.^20^ In Italy, he was awarded the rank of Lieutenant-Colonel and was the chief consultant in ophthalmology to the Allied army in the Mediterranean.^12^ For a man with such an extraordinary life, the war provided him with another epic episode of his already colourful story.

On his way to North Africa, aboard the hospital ship *HMS Windsor Castle* Benjamin came under enemy fire on the night of the 23^rd^ March 1943. The *Windsor* Castle was hit by a torpedo and sank 30 miles off Oran, Algeria. Benjamin was luckily rescued in his pyjamas by the destroyer *HMS Eggesford* but he never forgot the hours he spent waiting for rescue in the turbulent sea and the manic chaos that preceded it. Later in life, he made a private pilgrimage to the village of Eggesford in Devon to give thanks for his rescue.^12^ It is apt that Benjamin served on a ship named after his most famous local landmark as he lived within walking distance of the actual Windsor Castle. In the latter half of the war, Benjamin published a noteworthy book called ‘*A Manual for Field Officers*’.^12^ For his military service and, specifically, his advisory role to the Allied Mediterranean Command, Benjamin was awarded an OBE upon the war's closure.^12^

The war ended in 1945 and Benjamin was able to return swiftly back into civilian life and clinical practice. Many of his old patients had been impatient for his return and were quick to sign up once they knew his military service had ended. Upon returning to England, Benjamin was appointed consultant ophthalmic surgeon to Park Prewitt Emergency Medical Service Hospital near Basingstoke and to the Canadian War Memorial Hospital in Taplow both close to where the Rycrofts lived.^27^ In the post-war years, British healthcare underwent a profound change with the creation of the National Health Service in 1948. Despite his humble origins, Benjamin was surprisingly a sceptic of the socialised model. Benjamin believed it to be illogical and his efficiency-focused mind meant he remained unconvinced by the service. Despite this, Benjamin adapted well to the change and never forcibly opposed the new nationalised system.^12^

## Queen Victoria hospital, East Grinstead and corneal donation 1947–1959

The great turning point in Benjamin's entire career came not long after the war as he was invited by the renowned plastic surgeon Sir Archibald McIndoe (1900–1960) to come and work at the Queen Victoria Hospital in East Grinstead, Sussex.^27^ The hospital was already famed for its ground-breaking plastic surgery on burned and mutilated veterans but McIndoe wanted Benjamin to establish a much-needed eye department within the unit.^33^ Many of the veterans had corneal trauma in addition to the burns on their bodies so McIndoe naturally turned to the nation's leading expert on keratoplasty. This was the opportunity that Benjamin had been waiting for and using all his knowledge and expertise he was able to launch the hospital's Corneo-Plastic Unit in 1947.^33^ The unit would routinely perform corneal grafts, lacrimal surgery, eyelid surgery and socket surgery. Benjamin was at the forefront of most of this work. Benjamin and his unit developed such a strong reputation that students and admirers would travel from all over the world to witness the ‘magic’ that was being performed in the eye theatres.^12^ The strength of the Queen Victoria Hospital lay in its modest size and closely knit team. This meant that the training was impeccable with the surgical team operating as a well-oiled machine under Benjamin's command due to the many hours of practice together with few staffing changes.^12^ The reputation of the unit grew considerably which led to surgical proficiency being further enhanced as it became more competitive to work and study at the hospital under Benjamin's tutelage.^33^

If Benjamin had retired now, then he would have already left a long-lasting legacy in his specialist field of keratoplasty but his greatest impact in British ophthalmology came in 1952.^12^ The recurrent problem throughout most of Benjamin's career was the simple issue of insufficient donors. Benjamin had the publicity and skills but lacked the corneas to carry out keratoplasty on a large scale. The law then in place was the archaic Anatomy Act of 1832. This restrictive law meant a person's eye could only be used if the body had been left at a medical school for dissection.^33^ On the 11^th^ of December 1951, a meeting was held at the hospital with politicians from all major parties attending to hear Rycroft's and McIndoe's passionate plea for more donor eyes.^33^ Benjamin led this meeting and was in his element explaining how waiting lists were continuously growing as a result of the donor shortage. This meeting started a successful campaign that would go on to radically change the way organ donation was carried out in the UK. Named the ‘Sight for the Blind’ campaign it worked with various voluntary organisations to distribute information and identification cards to people who wanted to donate their eyes for grafting once they had died. A concurrent radio and television series were also created to help raise awareness. The campaign inspired significant publicity for the endeavour to increase access to corneal grafting and ultimately a bill was passed by Parliament in May 1952.^33^ The bill removed many of the complicated legal issues that had held donors back and also established Eye Banks to store donated eyes. The first British Eye Bank was set up at the Queen Victoria Hospital and was until the 1980s the only one in the UK and became the first to be founded outside of the USA.^34^ There the famed ophthalmologist Richard Townley Paton (1902–1984) founded the world's first eye bank in 1944.^35^

Staff would later recall their excitement at the first train arriving in East Grinstead with dozens of donor's eyes on-board ready to be used.^12^ The then-president of the Royal College of Surgeons, Sir Cecil Wakeley (1892–1979) used his hefty support to help Benjamin's campaign as did the South East Regional Hospital Board, the press and many other prominent ophthalmologists. But it was ultimately Benjamin alone who deserves the greatest credit for its success as he led the campaign from the front and became the face of the movement.^12^ As a result of this landmark act, Benjamin and other ophthalmologists were able to perform keratoplasty on a large scale for the very first time.^27^ What adds to this triumph is that in 1961 the 1952 act was further expanded to include other human tissue and is widely considered to be an early precursor of today's Human Tissue Act (2004). One could, therefore, argue that Benjamin Rycroft was the originator of legislation for collecting, preserving and using human tissue for transplants in the UK. Thousands of people each year are on the waiting list for transplants and thus owe gratitude to the work of Benjamin Rycroft.^12^ Benjamin's reward for his contribution was to be appointed clinical director of the Pocklington Eye Transplantation Centre at the Royal College of Surgeons.^15^ Benjamin had been pivotal in securing the funding to set up the Pocklington unit.

In 1953 and 1954, Benjamin published further papers on his theories regarding the novel idea of sterilising donor eyes pre-operatively.^36^ Benjamin believed that a paraffin suspension using both the recently discovered penicillin and streptomycin powder would aid the sterilisation of the donated materials.^36^ Benjamin decided to further his literary portfolio in 1955 by publishing and editing a book inventively titled *Corneal Grafts.*^12^ The book was a thorough analysis and overview of the titular subject and four of the sixteen chapters were authored by Rycroft.^12^ He managed to attract many other leading ophthalmological authorities from around the world to contribute chapters to the book which became the first of its kind to be published in English.^27^ The book was incredibly well received by the ophthalmological community and revealed Benjamin to be a talented and engaging writer. For many years, this book was seen as the go-to guide on the subject matter. By this point in his career Benjamin had published, either alone or collaboratively eleven journal articles on corneal grafts.^12^

## Later career 1959–1967

As Benjamin entered the twilight stages of his career, some might have expected him to slow down and ease his work but that was far from the case. In 1959, he hosted the First Corneo-Plastic Conference in East Grinstead which was likely the first of its kind worldwide.^12^ The event was unique in its specific subject matter. The conference was duly funded by willing patients and businessmen who were all devotees of Benjamin.^12^ The event was a huge success and attendees came from not just around the UK but also abroad. Benjamin viewed the conference as one of the highlights of his career and was always keen to try and host another event in the future. Having spent his entire working life committed to advancing healthcare and improving the lives of his beloved patients, Benjamin's greatest honour came in 1960 when he was knighted by Elizabeth II.^27^ This was in recognition of not just his long career but also his surgical innovations, teaching, leadership, academic writing, exemplary war service, donor campaigning and gratitude from his patients.

Throughout the early 1960s, Benjamin's reputation was at its pinnacle. In addition to all his other positions and titles, he was made President of the Section of Ophthalmology of the Royal Society of Medicine from 1960 to 1961.^15^ Benjamin was now entering the final stages of his career, so his clinical work began to inevitably slow, albeit modestly, in an effort to create slightly more freedom. This enabled him to travel and lecture abroad with Mary, as always, trying her hardest to join him on his voyages. He went on many lecture tours and was honoured by members of the ophthalmological societies of Australia, New Zealand, South Africa, France, Italy and Belgium.^18^ In 1964, the Sultan of Brunei even conferred on him the Most Blessed Order of Stia Negara Brunei.^18^ This unusual honour suggests that Benjamin may have treated the Sultan or a member of his family at some point in the preceding years.

By this point in his career, Benjamin and Mary were able to indulge themselves in the beauty of Bishop's Lodge (Berkshire). Benjamin adored the countryside life and his most prized possession was his herd of Jersey cattle which he cared for in the same ways he cared for his patients. Benjamin transferred many of his medical skills and roles over to the agricultural world. He was a member of the Royal East Berkshire Agricultural Association and became known for his wisdom and teaching skills, regularly training new and young farmers in animal husbandry and farming skills.^27^ He was the face of numerous local garden shows, rode horses to a show standard, hunted and took up fly fishing.^27^ Benjamin's favourite flowers were roses which he cultivated in excess and he would tend to them daily on his way home from the hospital. The proximity to Windsor was beneficial for Benjamin as he became a lay officer of the medieval St George's Chapel within the royal Windsor Castle.^12^ Benjamin loved to show overseas tourists the wonders hidden in the chapel with most guests not knowing that their tour guide was one of the world's most respected ophthalmologists. The chapel provided him with another opportunity to play his beloved organ.

In 1965, Benjamin served in another distinguished role abroad when he was appointed honorary president at the Fourth International Course of Ophthalmology at the Barraquer Institute in Spain.^12^ The Barraquer family is a Spanish ophthalmological dynasty which has devoted 150 years to Spanish eyecare.^37^ The family has produced generations of notable ophthalmologists and in 1941 they established the first dedicated eye centre in Spain, in Barcelona. In 1947, they then set up a separate Barraquer Institute which focused on ophthalmological research, teaching, awareness and the promotion of the subject. Nowadays the institute is affiliated with the Autonomous University of Barcelona [AUB]. The Barraquers were, such as Benjamin, keratoplasty enthusiasts and followed Benjamin's lead in establishing their own Eye Bank in 1962, the first in continental Europe.^37^ Benjamin was a close family friend of the Barraquer's which is why they honoured him at their conference. It was likely at this conference that Jose Barraquer (1916–1998) described his recent landmark operation of a hinged flap posterior lamellar keratoplasty.^35^ Upon his return to England, Benjamin fell ill which meant he was unable to travel to Chicago in the US where he was due to be the keynote speaker at the prestigious Chicago Ophthalmological Society.^12^

Luckily, Benjamin was able to recover from this illness and was fit enough to give the Doyne Memorial Lecture before the Oxford Ophthalmological Congress in July 1965.^12^ He chose the topic ‘The Corneal Graft-Past, Present and Future’. Attendees particularly enjoyed the early part of his talk when he addressed the history of corneal grafting which was something Benjamin had always found interesting. Benjamin would claim that it was a British surgeon Sir Astley Cooper (1768–1841) who actually inspired the German Franz Reisinger (1768–1855) to carry out the first corneal transplant on animals in 1818.^12^ Reisinger had apparently visited Cooper at Guy's hospital in 1817 and witnessed a demonstration of a primitive skin graft and decided to replicate a similar experiment on a cornea the following year. Reisinger is coincidentally the person who actually coined the term keratoplasty.^35^

Benjamin managed to visit the USA in the early months of 1966 to make up for his illness the year prior.^12^ By this point in his career, Benjamin was focusing more on his role as the clinical director of the Pocklington Eye Transplantation Research unit at the Royal College of Surgeons. He remained closely involved with the department at East Grinstead but found it hard to juggle all his prestigious commitments. He also continued to make sure he dedicated a large portion of his time and energy to the multitude of private patients who wanted to be treated by him.^12^

## Death and legacy

Having thoroughly enjoyed the First Corneo-Plastic Conference in 1959, Benjamin decided that the time was right to hold a second conference in the summer of 1967.^12^ Benjamin viewed this conference as the zenith of his career and presumably would retire not long after the event. He set to work preparing for the upcoming event, but God had other plans and, on the 29th of March 1967, the unsinkable Benjamin Rycroft died suddenly of a myocardial infarction at the age of 64.^12^ Despite a few minor episodes of poor health over the previous years, the death of Benjamin was a shock to many, leaving the ophthalmological world struck with grief. Benjamin's beloved St George's Chapel honoured Benjamin by hosting an emotional memorial service on the 2^nd^ of May.^33^

The Second Corneo-Plastic Conference still went ahead in 1967 at the Waldorf Hotel in London but was now seen as a tribute to Benjamin.^38^ Benjamin's widow Mary and son Peter remained heavily involved with the conference and attendees travelled from all over the world to pay their respect and show tribute to his influence on the eye.^38^ Members of the Barraquer family and the American ophthalmologist Townley-Paton were some of the more well-known guests. Benjamin was also due to be a speaker at the First South African International Ophthalmic Symposium in 1968 but his untimely death meant that talk never went ahead either.^38^

Benjamin was survived by his spouse Mary, who passed away in 1982, and his two sons Peter and Rodney. Peter followed his father Benjamin into the ophthalmological world.^39^ Peter studied at Trinity College Cambridge, St Bartholomew's and Moorfields, the latter two being places where his father had likewise studied.^40^ Peter was widely seen as Benjamin's heir apparent as he had over time taken on many of his father's former roles and many of Benjamin's private patients naturally turned to his son after Benjamin's death. The Rycroft's were again hit by tragedy the following year as Peter was involved in a fatal motor accident in January of 1968.^40^ Peter did not survive the crash with his son Andrew being the only survivor.^40^ The ophthalmological world was again hit hard by the death and the condolences and sympathies for the Rycroft family flooded in.

## Conclusion

Benjamin is remembered for his boundless energy and sociable nature.^41^ These traits he poured into an impeccable war service record and an enviable ophthalmological career. In the 1960s and 1970s, Sir Stewart Duke-Elder, Sir Harold Ridley and Sir Benjamin Rycroft were widely viewed as the three wise men of British ophthalmology. The reputation of Ridley is deservedly the greatest given that his cataract surgery has restored sight to over 200 million people worldwide.^42^ Sadly, Rycroft's name has largely vanished from popular medical memory today. That may be due to being eclipsed in his association with the Queen Victoria Hospital by famous plastic surgeons such as Sir Archibald McIndoe. It may also be because Rycroft's humble beginnings afforded him fewer opportunities, for example, by entering ophthalmology at a later age after labouring in general practice. His early death compared to his contemporaries may have also been a factor. Despite this, Benjamin's legacy lives on through his contribution to advancing keratoplasty and the introduction of organ donor register.

Benjamin did not invent keratoplasty nor was he the first Briton to perform the operation but his work with organ donation made him the pivotal person in enabling it to be carried out on a large scale for the first time.^30^ He took an operation performed on a few dozen to the masses and his work means that today there are roughly 4000 corneal grafts carried out annually in the UK.^43^ He did not realise it then, but his organ donation campaign would overshadow his surgical skills and become his greatest legacy. This is because his organ donation reform continues to save thousands of lives in the 21st century and not just for ophthalmological patients but also those who receive other transplants. Many organ transplant receivers would be grateful for Benjamin's work but, sadly, are unaware of his existence. We hope that this article goes some way to remembering and recording the achievements of Sir Benjamin Rycroft.
